# Acute left ventricular insufficiency in a Burkitt Lymphoma patient with myocardial involvement and extensive local tumor cell lysis: a case report

**DOI:** 10.1186/s12872-022-02480-5

**Published:** 2022-02-04

**Authors:** Maren Schmiester, Eva Tranter, Alessandro Lorusso, Florian Blaschke, Dominik Geisel, Lars Bullinger, Frederik Damm, Il-Kang Na

**Affiliations:** 1 Department of Hematology, Oncology, and Tumor Immunology, Charité-Universitätsmedizin Berlin, Corporate Member of Freie Universität Berlin and Humboldt-Universität zu Berlin, Berlin, Germany; 2 Department of Cardiology, Charité-Universitätsmedizin Berlin, Corporate Member of Freie Universität Berlin and Humboldt-Universität zu Berlin, Berlin, Germany; 3 Department of Radiology, Charité-Universitätsmedizin Berlin, Corporate Member of Freie Universität Berlin and Humboldt-Universität zu Berlin, Berlin, Germany

**Keywords:** Burkitt Lymphoma, Case report, Cardiac neoplasms, Myocardial lymphoma involvement, Acute cardiac insufficiency

## Abstract

**Background:**

Burkitt lymphoma (BL) is a rare disease with the sporadic variant accounting for less than 1% of adult non-Hodgkin lymphomas. BL usually presents with an abdominal bulk, but extranodal disease affecting the bone marrow and central nervous system is common. Cardiac manifestations, however, are exceedingly rare, with less than 30 cases reported in the literature.

**Case presentation:**

We report on a 54-year-old male patient with a six week-long history of paranasal sinus swelling, fatigue and dyspnea on exertion. Stage IV sporadic BL with extensive lymphonodal and cardiovascular involvement was diagnosed. Manifestations included supra- and infradiaphragmatic lymphadenopathy as well as infiltration of the aortic root, the pericardium, the right atrium and the right ventricle. EBV-reactivation was detected, which is uncommon in the sporadic subtype. After initial full-dose chemotherapy with very good BL control, the patient developed acute, but fully reversible cardiac insufficiency. Myocardial lymphoma involvement receded completely during the following two therapy cycles, while cardiac function periodically deteriorated shortly after chemotherapy administration and quickly recovered thereafter. Interestingly, the decline in cardiac function lessened with decreasing myocardial lymphoma manifestation. Once the cardiovascular BL infiltration was resolved, cardiac function remained stable throughout further treatment. Following seven cycles of chemotherapy and mediastinal radiation, the patient is now in continued complete remission.

**Conclusions:**

Although rare, cardiac involvement in BL can quickly become life-threatening due to rapid lymphoma doubling time and should therefore be considered at initial diagnosis. This case suggests an association between myocardial infiltration, chemotherapy associated tumor cell lysis and transient deterioration of cardiac function until the damage caused by the underlying lymphoma could be restored. While additional studies are needed to further elucidate the mechanisms of acute cardiac insufficiency due to lymphoma lysis in the infiltrated structures, prompt BL control and full recovery of the patient supports courageous treatment start despite extensive cardiovascular involvement.

## Background

Burkitt lymphomas (BL) are rare, highly aggressive hematologic neoplasms accounting for less than 1% of all non-Hodgkin lymphomas (NHL) [1]. They are characterized by a marked proliferative activity as well as translocation and constitutive activation of the MYC oncogene. Three distinct clinical variants have been described, with the endemic form occurring in association with Epstein-Barr-Virus (EBV) infection in the equatorial regions of Africa, the immunodeficiency-associated form occurring as a manifestation of HIV infection and the sporadic form occurring non-endemically globally. While EBV-association is found in 95% of endemic cases, it is very uncommon in sporadic BL [2]. Payer’s plaques of the ileocecal region are the most frequent site of origin in this subtype, resulting in an abdominal tumor [3]. Cardiac manifestations, on the other hand, are extremely rare [4]. Indeed, there are less than 30 reports published in the English literature as of now. In the few case studies available, more than a third of affected patients died of the disease within the first days after diagnosis [5], underscoring the need for swift diagnosis and treatment. Interestingly, the incidence of cardiac involvement across all NHL types appears much higher with reported ranges of 10–20%. This affectation is often only discovered on autopsy, suggesting a lesser clinical relevance in these lymphomas as compared to BL [6–8].

Here, we present the case of a patient with BL who displayed extensive cardiovascular manifestations and a rapidly progressing disease with a life-threatening situation at the time of diagnosis. During chemotherapy, the patient experienced transient acute cardiac insufficiency, perhaps due to myocardial tumor cell lysis. Cardiac function fully recovered alongside with chemotherapy induced tumor control.

## Case presentation

An otherwise healthy 54-year-old Caucasian male presented to our clinic with a six week-long history of paranasal sinus swelling, fatigue and dyspnea on exertion. Fever and weight loss were denied. Additional relevant findings on physical examination included tachycardia of 110/min and muffled heart sounds. On room air, oxygen saturation was normal (97%) and respiratory rate was not elevated. No spleno- or hepatomegaly was palpable. Left-sided submandibular lymph node enlargement was present, other lymph nodes were not enlarged. Discrete congestion of cervical veins was seen, with the right side being more prominent. Slight bilateral ankle edema was noted. Neurological examination showed no abnormalities. Eastern Cooperative Oncology Group (ECOG) performance status was estimated at 2 [9].

Relevant laboratory findings included an elevated lactate dehydrogenase level (LDH) of 620 U/l (reference < 250 U/l) and EBV copies of 395,000/ml. Testing for HIV was negative. A 12-lead electrocardiogram showed low QRS voltages and sinus tachycardia.

**Fig. 1:**
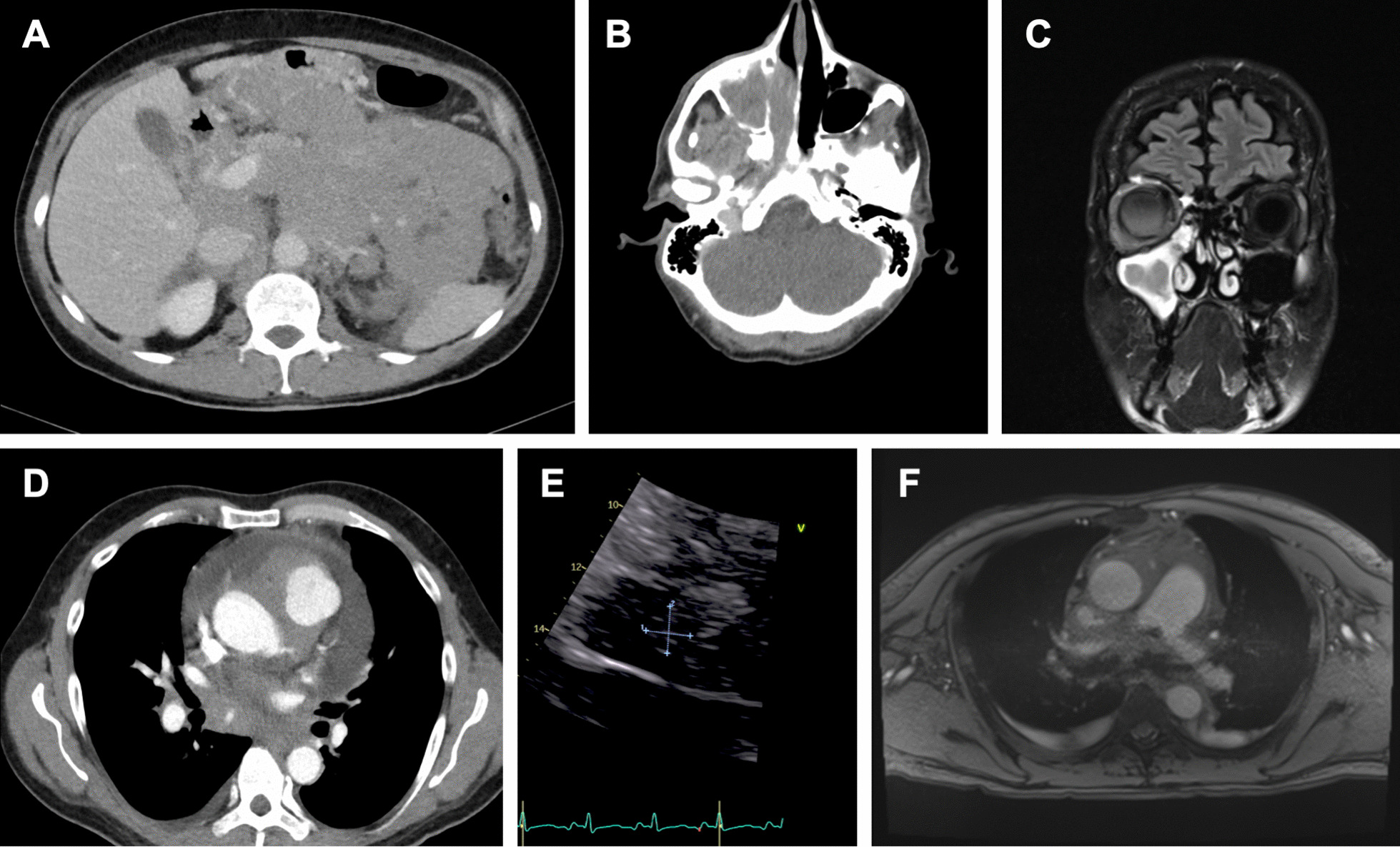
CT and MR imaging reveal extensive BL manifestations. **A** Contrast-enhanced CT, large abdominal manifestation with infiltration of pancreas and mesenteric root. **B** and **C** Contrast-enhanced skull CT and MRI, destruction of posterior wall of right maxillary sinus by BL manifestation. **D** Contrast-enhanced chest CT, mediastinal manifestation with infiltration of supraaortic vessels. **E** TTE revealing a solid mass of 2.2 × 1.3 cm of the right atrium. **F** Cardiac MRI: mediastinal and pericardial mass with infiltration of pericardium and bilateral pleural effusion

Computed tomography (CT) scans of the head and neck, chest, abdomen and pelvis showed cervical, mediastinal, paraaortic and retroperitoneal lymphadenopathy as well as an infiltration of the frontal, maxillary and sphenoidal sinuses by a suspected lymphoma. Notably, the mediastinal adenopathy showed encasement of the aortic root, the coronary arteries and an infiltration of the pericardium, the right ventricle and the right atrium (Fig. 1A–D).

An initial transthoracic echocardiography (TTE) revealed a hemodynamically relevant, circumferential pericardial effusion of about 2 cm with consecutive diastolic collapse of the right atrium. Left ventricular ejection fraction (LVEF) was normal at 60%. The assessment of the right ventricular (RV) function was compromised by pericardial effusion; longitudinal function was normal (tricuspid lateral annular peak systolic velocity 0.23 m/s, tricuspid annular plane systolic excursion 23 mm) and the proximal wall of the right ventricular outflow tract (RVOT) showed a thickening of > 1 cm with diminished local contractility. In addition, a solid right atrial mass of 13 × 22 mm was seen, and possible differential diagnoses of intra-cardiac lymphoma, thrombus or myxoma were discussed (Fig. 1E).

Pericardiocentesis allowed the drainage of 1200 ml of effusion over the course of three days. Cardiovascular magnetic resonance imaging was performed to analyze the extent of cardiac involvement in more detail. Here, pericardial and myocardial infiltration of the right atrium and ventricle and infiltration of the aortic root and was confirmed (Fig. 1F). The etiology of the right atrial mass remained unclear. As the risk of severe bleeding under chemotherapy and extensive vascular infiltration was assessed as relevant, only low-dose anticoagulation with low-molecular-weight heparin was initiated.

Biopsies were obtained from the paranasal sinuses, a submandibular lymph node and the bone marrow. A spinal tap was also performed, which showed no pathological findings. The patient was started on a prephase treatment with prednisone (100 mg/day for 5 days) and cyclophosphamide (200 mg/m^2^ for 3 days).

**Fig. 2:**
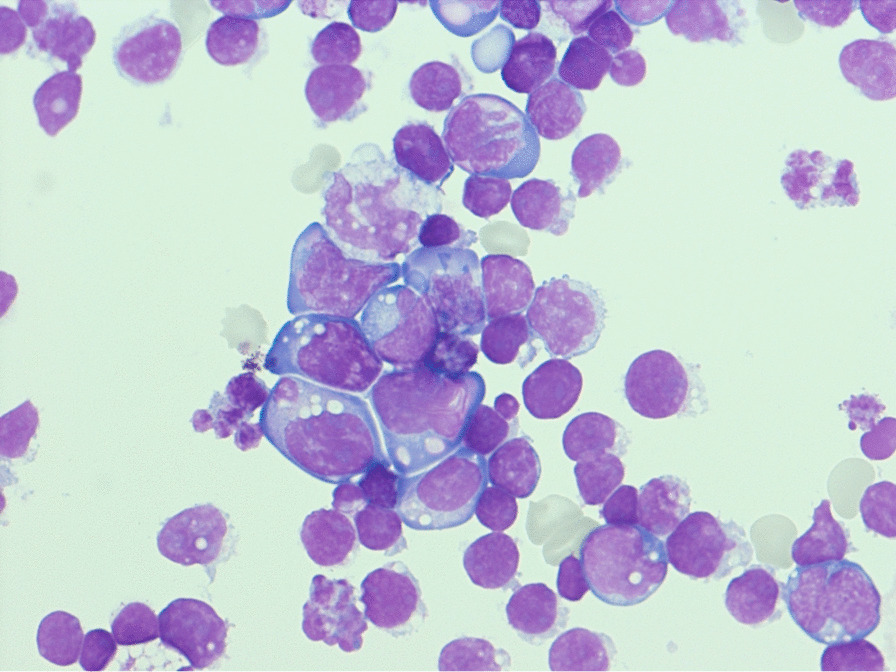
Morphologic analysis of the pericardial effusion. Typical medium sized Burkitt lymphoma cells with cytoplasmic vacuoles

Morphologic (Fig. 2) and flow cytometric analysis of the pericardial effusion described a mature B-cell neoplasia with an immunophenotype of CD79b^+^, CD19^+^, CD20^+^, CD22^+^, CD10^+^, CD23^+^ indicative for an aggressive B-cell lymphoma. Based on this preliminary diagnosis, prephase treatment was escalated to R-CHOP due to the extent of disease and to treat the EBV reactivation (rituximab 375 mg/m^2^ administered twice at an interval of one week, cyclophosphamide 150 mg/m^2^ in order to reach a complete dose of 750 mg/m^2^, doxorubicin 50 mg/m^2^ and vincristine 1.4 mg/m^2^).

Therapy was well tolerated. However, a follow-up TTE three days after administration revealed a sudden drop in LVEF to 35–40% with LV dilatation, akinesia of the apical and medial septal zones and hypokinesia of the medial segments. The right atrial mass was still present, and the RV showed normal contractility. The patient reported dyspnea on exertion, but no chest pain. Troponin T concentration was mildly elevated at 34 ng/l (reference < 14 ng/l). Invasive cardiac catheterization was not performed in light of the atypical constellation of findings for acute myocardial infarction and a high likelihood of a lymphoma and/or lymphoma therapy associated cause of the symptoms. Nevertheless, a coronary computed tomography angiography was performed, which showed no evidence for coronary artery stenosis. Subsequently, therapy with a ß-blocker and an angiotensin-converting enzyme (ACE) inhibitor was initiated.

In the following days, immunohistochemical examination and fluorescence in situ hybridization (FISH) of the biopsy material from the submandibular lymph node/paranasal sinuses led to the diagnosis of BL with cells displaying a proliferation rate of virtually 100%, an immunophenotype of CD20^+^, CD10^+^, BCL6^+^, MYC^+^, TdT^−^, BCL2, a strong positivity for a EBV and a rearrangement of *c-MYC (Chr 8q24)* on FISH analysis. There was no BL manifestation in the bone marrow. On day 10 following start of therapy, the patient was discharged with the diagnosis of a BL stage IV disease in a clinically much improved state (ECOG 1).

Chemotherapy was then continued analogous to block A1 of the German Multicenter Study Group on Adult Acute Lymphoblastic Leukemia (GMALL) B-ALL/NHL protocol as recommended by the local tumor board [10]. Interestingly, prior to treatment start the LVEF had normalized to 60% and the right atrial mass was no longer detectable on TTE, suggesting that the transient cardiac deterioration was indeed most likely attributable to the BL manifestation, which had regressed after R-CHOP therapy. We therefore decided to continue with the full dose of the A1 therapy block (rituximab 375 mg/m^2^, vincristine 2 mg, cytarabine 150 mg/m^2^ twice daily for two days, etoposide 100 mg/m^2^ for two days, methotrexate (MTX) 1500 mg/m^2^, ifosfamide 800 mg/m^2^ for five days and dexamethasone 10 mg/m^2^ for five days; intrathecal prophylaxis with cytarabine 40 mg, dexamethasone 4 mg and MTX 15 mg was administered twice) while continuing to closely monitor cardiac function. After the second chemotherapy block, TTE again revealed a slight decrease in LVEF (45–50%) with reduced longitudinal strain.

A follow-up TTE during the third cycle of chemotherapy showed a normal LVEF with borderline longitudinal strain and normal RV function. Notably, the thickening of the RV wall had vanished in comparison to the baseline exam.

Overall, the patient was treated with accumulating doses of chemotherapy according to protocol in a total of 7 cycles, and the LVEF remained normal after the initial recovery from acute dysfunction. CT staging revealed a complete remission after completion of chemotherapy. Subsequently, the patient underwent consolidating radiation of the mediastinum at a cumulative dose of 36 Gy in 13 fractions.

With a follow-up of 16 months after diagnosis, the patient is in continuous complete remission as of now.

## Discussion and conclusions

BL is one of the most aggressive B-cell lymphomas with a rapid doubling time [1]. The case presented here is very unusual regarding its EBV-reactivation in a sporadic BL and its clinical presentation with extensive cardiac involvement.

EBV reactivation, as seen in our patient, is rarely seen in sporadic BL and much more frequent in the immunodeficiency-associated form [11]. However, negative testing for HIV ruled out acquired immune deficiency syndrome (AIDS) as a reason for immunosuppression and CD21-negativity favors the diagnosis of a sporadic BL form.

In 2016, Chan et al. published a review of all 22 cases of patients presenting with intracardiac BL manifestations reported in the English literature, and we found six additional cases that have been published since then. Patients were predominantly male and presented with similar symptoms as our patient, including fatigue, tachycardia and dyspnea. Information on administered chemotherapy is available for 18 cases. While about 2/3 of patients receiving treatment responded to therapy, the rest died within days to weeks after diagnosis, with heart failure being a major contributing factor to the causes of death. On the other hand, all patients reported not to have received therapy died of the disease within days [5, 12–17]. Thus, based on the very limited data available, rapid initiation of treatment—at the risk of severe acute cardiac insufficiency—seems to be the only potentially lifesaving option for affected patients. It remains uncertain whether an increased susceptibility to therapy-induced myocardial toxicity or intracardiac tumor cell lysis during treatment is responsible for cardiac failure.

Acrolein, a metabolite of cyclophosphamide, is known to have a direct toxic effect on endothelial cells and the myocardium [18]. Anthracyclines can also induce acute and late cardiotoxicity due to increased sympathetic stimulation, causing microvascular dysfunction as well as alterations in cytokine, free radical and catecholamine receptor levels. Acute toxicity usually manifests as disturbance in electrical conduction, while chronic toxicity can lead to reductions in LVEF [19–22]. While there is no universally-accepted clinical definition of chemotherapy-associated cardiomyopathy, general consensus entails a reduction of global LVEF > 10–15% [23].

The localized left ventricular akinesia observed in our patient shortly after initial R-CHOP therapy is therefore not typical for acute chemotherapy-induced toxicity. Furthermore, growing evidence suggests that low troponin levels after anthracycline therapy—as observed in our patient—are related to better cardiac outcomes due to limited myocardial injury [24]. We therefore believe that rapid intracardiac tumor lysis, rather than myocyte loss, mainly contributed to the decrease in LVEF in our patient, perhaps through to changes in the cardiac microcirculation and in energy metabolism. The sudden and successively smaller drops in LVEF observed periodically after the following chemotherapy cycles appear related to the amount of residual intracardiac lymphoma mass, culminating in a complete recovery of LVEF.

Interestingly, imaging at diagnosis showed predominant BL manifestations in the right heart, while the observed localized cardiac malfunction manifested in the left ventricle. We believe that BL infiltration too small to be picked up on CT or echocardiography was also present in the left ventricle, explaining these discrepancies. However, to elucidate the underlying mechanisms of acute cardiac insufficiency in patients with cardiac tumor involvement, preclinical studies are needed to explore possibilities beyond therapy-associated toxicity.

In conclusion, in the rare case of cardiovascular involvement secondary to BL, rapid lymphoma control is crucial to prevent life-threatening hemodynamic instability. This case illustrates that a prompt treatment start can save affected patients’ lives with full cardiovascular recovery despite transient deterioration of cardiac function.

## Data Availability

The data analyzed are available from the corresponding author on reasonable request.

## Abbreviations

ACE: Angiotensin-converting enzyme
B-ALL: B-lymphocytic acute lymphoid leukemia
BCL2: B-cell lymphoma 2
BCL6: B-cell lymphoma 6
BL: Burkitt Lymphoma
CD: Class of differentiation
CT: Computed tomography
EBV: Epstein Barr Virus
ECOG: Eastern Cooperative Oncology Group
FISH: Fluorescence in-situ hybridization
GMALL: The German Multicenter Study Group on Adult Acute Lymphoblastic Leukemia
HIV: Human immunodeficiency virus
LDH: Lactate dehydrogenase
LVEF: Left ventricular ejection fraction
MTX: Methotrexate
NHL: Non-Hodgkin’s Lymphoma
R-CHOP: Chemotherapy regimen containing Rituximab, Cyclophosphamide, Doxorubicin, Vincristine and Prednisone
RVOT: Right ventricular outflow tract
TTE: Transthoracic echocardiogram
